# Is Continued Improvement After Automated Virtual Reality Exposure Therapy for Spider Phobia Explained by Subsequent *in-vivo* Exposure? A First Test of the Lowered Threshold Hypothesis

**DOI:** 10.3389/fpsyt.2021.645273

**Published:** 2021-05-20

**Authors:** Philip Lindner, Peter Dafgård, Alexander Miloff, Gerhard Andersson, Lena Reuterskiöld, William Hamilton, Per Carlbring

**Affiliations:** ^1^Department of Psychology, Stockholm University, Stockholm, Sweden; ^2^Centre for Psychiatry Research, Department of Clinical Neuroscience, Karolinska Institutet & Stockholm Health Care Services, Region Stockholm, Stockholm, Sweden; ^3^Department of Behavioral Sciences and Learning, Linköping University, Linköping, Sweden; ^4^Department of Biomedical and Clinical Sciences, Linköping University, Linköping, Sweden; ^5^Mimerse, Stockholm, Sweden

**Keywords:** exposure therapy, virtual reality, specific phobia, adherence, long-term

## Abstract

Consumer Virtual Reality (VR) technology offers a powerful, immersive medium for scalable dissemination of mental health interventions. Decades of research has shown VR exposure therapy to be efficacious in the treatment of anxiety disorders and that the fear reduction generalizes to real-world stimuli. Many studies also report continued improvement over time, after discontinuing VR use. The lowered threshold hypothesis states that this continued improvement is moderated by lowering the threshold to conduct subsequent *in-vivo* exposure. The current study is the first to formally test this hypothesis, using data from a recent trial on automated VR exposure therapy for spider phobia, in which participants (*n* = 49) were followed for 1 year, completing assessments 1 week, 3 and 12 months post-treatment. The assessment included validated self-report of phobia symptoms, a standardized behavioral approach test featuring a real spider, and a questionnaire for self-reporting frequency of *in-vivo* exposures since last assessment. Number of *in-vivo* exposures was found to be independently associated with greater symptom decrease in longitudinal outcome models. In sequential structural equation models, immediate post-treatment symptom reduction was associated with subsequent *in-vivo* exposures, which in turn was associated with continued symptom reduction. However, this applied only to self-reported phobia symptoms (not behavioral avoidance) and no associations were found past 3 months. Our findings offer preliminary, partial support for the lowered threshold hypothesis, suggesting that VR exposure interventions may benefit from including explicit *in-virtuo* to *in-vivo* transitioning components.

## Introduction

Virtual Reality (VR) refers to technology capable of generating immersive experiences of being present in virtual, computer-generated environments, typically achieved through the use of a head mounted display that withholds the outside world, continuously tracks head rotation (and possibly position), and updates the stereoscopic display as the user looks around and interacts with environment (1). Although recognizable VR technology first appeared in the 1960's, it is only in the last 5 years that consumer VR devices have become common (standalone versions of which now cost only a few hundred USD), alongside mature ecosystems for development and dissemination of VR applications (2). This presents a paradigm shift also for clinical and public health applications of VR (3), numerous successful examples of which have accumulated in the last 25 years (4).

One such example is VR exposure therapy: here, VR technology is used to create and present virtual equivalents of phobic stimuli (e.g., animals, heights, and situations) to users in an immersive way (5, 6), to allow for graded, controlled, and systematic exposure until the fear response habituates and inhibitory learning occurs (7). This treatment format is attractive to both patients (8–10) and therapists (11, 12), is feasible also with adolescents (13)—although considerably less studied with this population (14)—and is associated with low rates of deterioration, i.e., unlikely to have negative effects (15). Recent research has shown that VR exposure can be self-directed (16), and even packaged as automated interventions relying on gamification components rather than a real-life therapist directing treatment (17–20), and even a virtual therapist (21, 22).

As VR continues to grow and establish itself as a consumer technology (23), automated and self-directed interventions have the potential to make a significant public health impact by addressing the large treatment gap (24) and delay in treatment-seeking for anxiety disorders (25), phobias in particular. Although some methodological concerns have been raised (26), meta-analyses have demonstrated efficacy of VR exposure therapy for anxiety disorders (27–29), even when only considering trials featuring *in-vivo* behavioral approach tests as outcomes (30). This latter finding reveals that a reduced fear response to virtual phobic stimuli generalizes to the *in-vivo* equivalents that users will ultimately face in real life. This generalization likely explains why many trials report continued symptom improvements at follow-ups 1 to 6 years after completing VR exposure therapy (18, 31–34). Notwithstanding the immediate fear reduction (30), the full clinical effect may come from lowering the threshold for users to subsequently engage in *in-vivo* exposure opportunities as they appear in everyday life, breaking the vicious circle of avoidance that maintains anxiety disorders (35).

Although the idea that post-treatment, continued exposure predicts continued improvement likely applies equally well to traditional exposure therapy (36), VR exposure therapy is unique in that here, continued exposure entails not only continuing to do what was taught in treatment, but doing it in a different way. Despite having been noted as a limitation of the extant literature (4, 17), the necessary transition from in-virtuo to *in-vivo* exposure—and implicitly the lowered threshold hypothesis—has received surprisingly little research attention. Indeed, we are not aware of any study that has measured and attempted to statistically model the moderating role of post-VR, *in-vivo* exposure in explaining long-term continued symptom improvement. Indirect evidence, however, comes from two trials featuring the same automated VR exposure intervention for spider phobia: in one trial (18), participants in the VR arm were given written and oral information explaining the rationale for transitioning to *in-vivo* exposure in everyday life, as well as instructions for planning, execution, and evaluating exposure tasks; while in the other trial (17), no such written material was provided. In line with the lowered threshold hypothesis, the former trial saw continued improvement during the follow-up period, while the latter did not. In another trial, completing *in-vivo* exposure exercises during an explicitly framed transition period following a single session of VR exposure therapy for public speaking anxiety, was independently associated with additional symptom decrease (16).

These findings warrant further investigation, since the lowered threshold hypothesis has important implications for the design of automated VR exposure interventions that show such a great public health potential. In the current study, using data from one of the trials on automated VR exposure therapy for spider phobia, and using complementary statistical modeling techniques, we report a first such investigation.

## Methods

### Ethics

The current study uses data from a randomized non-inferiority trial (18, 37) that was pre-registered (Clinicaltrials.gov: NCT02533310), received ethical approval from the Swedish Ethical Review Authority (2015/472-31 and 2015/1695-32), and had all participants provide written informed consent.

### Procedure and Participants

See the published trial protocol and primary report for details (18, 37). In brief, *n* = 100 participants were recruited from the general public, assessed for spider phobia, provided self-report measures, completed a standardized behavioral approach test (BAT) with a real spider (see Measures below). Included participants were randomized 1:1 to a single, 3 h session of either *in-vivo* exposure therapy (38), or a novel, automated, gamified VR exposure therapy intervention. Only participants in the VR arm (*n* = 50) were included in the current study, with all but one completing treatment. At the end of treatment, participants were given standardized oral and written material explaining the rationale for transitioning to *in-vivo* exposure in everyday life, as well as instructions for planning, execution and evaluating exposure tasks (39) (material available from the authors on request). One week (*n* = 49), 3 months (*n* = 45) and 1 year (*n* = 47) after receiving treatment, participants completed a similar assessment with the same outcome measures. Assessments were intended to be conducted on-site at Stockholm University; however, if a participant could not be scheduled for a visit, a mailed paper or online version of the included self-reports was made available. Across assessment occasions, a total of k = 34 assessments (of k = 191) were returned on paper or completed online, entailing that fewer BAT data points are available for analysis.

### Measures

For the current study, we selected the two outcome measures from the non-inferiority trial (18, 37) that showed the largest effect sizes, covering related by separated aspects of spider phobia: a standardized BAT used in previous research (40) providing an objective measure of avoidance behavior, as well as the self-rated Fear of Spider Questionnaire (FSQ) (41) covering a broader set of phobia symptoms. The BAT featured a real spider (a harmless species native to Sweden, ~2 cm in size including legs) and was scored in 13 steps (rated 0−12), with higher scores corresponding to lesser avoidance. The FSQ consists of 18 items, scored 1−7 for a maximum score of 126, with higher scores corresponding to more phobia symptoms.

**Figure 1:**
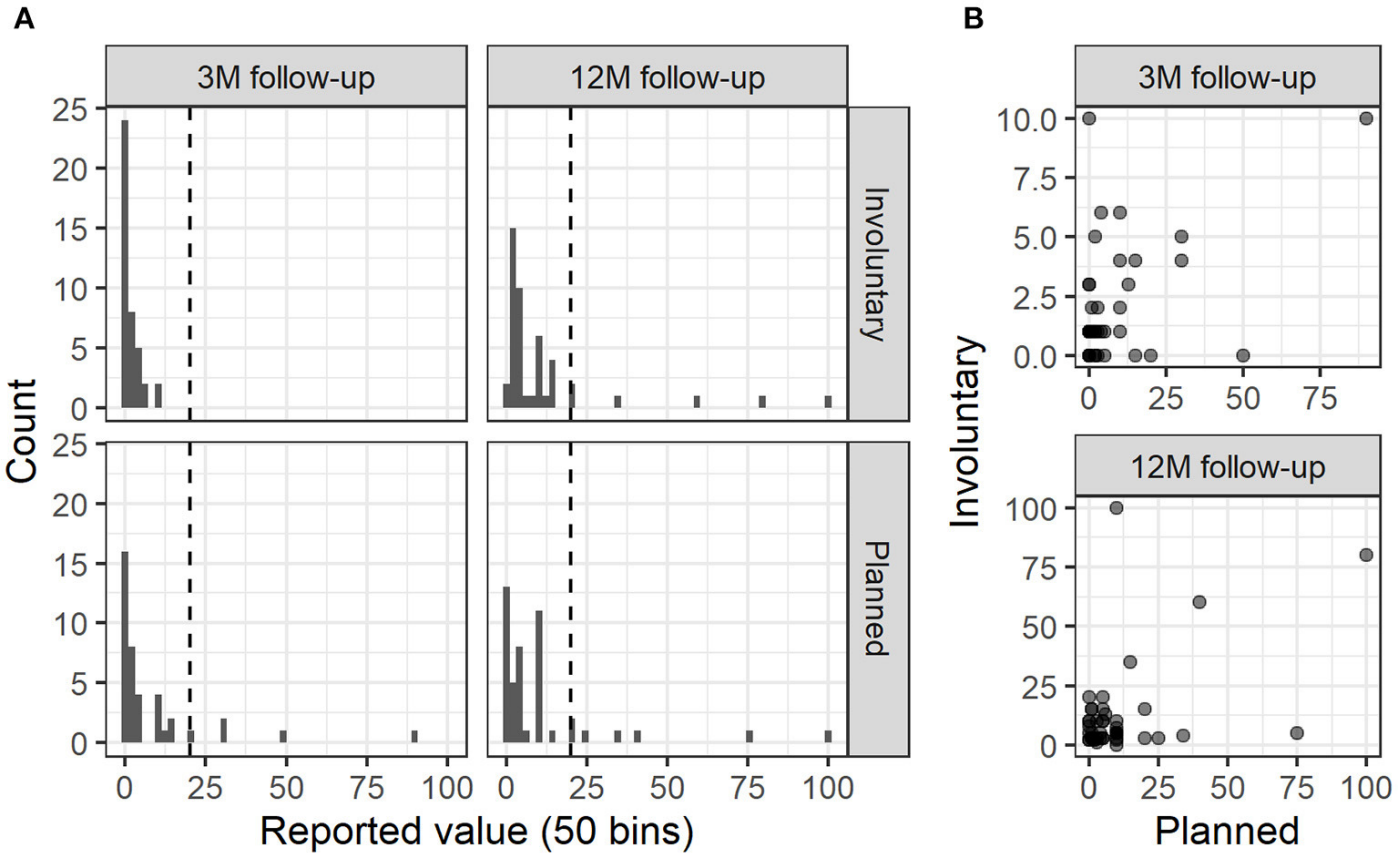
Distributions and correlations of planned and involuntary exposure occasions across time. **(A)** Distributions of (raw) reported exposure occasions split by type and assessment point. Dashed line shows chosen outlier threshold. **(B)** Scatterplots of planned vs. involuntary exposures split by assessment point.

At the 3 and 12 month follow-up assessments, participants also reported on extent of *in-vivo* exposure using a custom, standardized form. The form differentiated between planned exposure exercises vs. involuntary exposure to real spiders, and had participants estimate number of occasions since the last assessment. Exact phrasings used were “Approximately how many times have you deliberately and specifically for exposure purposes put yourself in a position where you were exposed to spiders,” and “Approximately how many times have you involuntarily been caught in situations where you were exposed to spiders?” (both translated from original Swedish). The raw reported values showed several outliers; see Figure 1 for distributions split by time points and type (Figure 1A), as well as a correlation scatter plot across time points (Figure 1B). Since there was no a prior reason to expect linear associations across this width of values (making raw values inappropriate), and in lieu of any established way of statistically handling outliers in this type of variable, we opted for an explorative approach wherein the initial analyses were repeated with three common outlier-adjustment alternatives, for each type of exposure and outcome (respectively). These adjustments involved either binarizing (zero or any), truncating outliers (any value >20, threshold chosen due to a sharp decrease across types and occasion as revealed by histograms), or omitting outliers (same threshold used in truncation). For sake of transparency, all models (including ones with the raw variable for comparison) are reported.

At the 3 month follow-up, the questionnaire also included an additional five questions on: whether they considered themselves to have actively continued to perform exposure exercises (Yes or No); whether they created a maintenance and transition plan as instructed (Yes or No), self-rated compliance to this plan (0−10, from Not at all to Completely); whether they believed that they had been given a good rationale on the need for maintenance (Yes or No); and whether they were given sufficient instructions for how to construct their plan (Yes or No).

### Analyses

For our initial test of the lowered threshold hypothesis, we ran cluster-bootstrapped general linear models (GLM) on each outcome (BAT or FSQ scores) and exposure type (planned or involuntary) separately, each with every version of the exposure frequency variable. All models featured a numeric time variable (post-treatment, 3 and 12 month follow-ups, scored as 0, 1, and 2), with exposure frequency as an additional, time-varying predictor (set to zero at post-treatment). Cluster-bootstrapped general linear modeling is a novel statistical technique, robust to normality assumptions, that is especially appropriate when there is risk of miss-specifying a corresponding mixed model (e.g., when few time points are available). Analyses were performed using the ClusterBootstrap R package (42), each with 10,000 repeats.

Second, the sequential and interactive associations captured by the lowered threshold hypothesis was examined by running structural equation models (SEM) on each outcome separately. Both models featured the truncated exposure frequency variable for several reasons: a numeric variable was necessary, results from the first analysis step suggested that this adjustment corrected for any measurement error, and this variable adjustment retained all available data, thereby maximizing power. In a longitudinal sequence, the SEMs used the score difference between two adjacent time points to predict the subsequent number of exposure occasions, which in turn predicted the next score difference, and so on. Covariance between adjacent score differences was also included. Robust Huber-White standard errors were calculated, and missing data estimated using maximum likelihood. Analyses were conducted using the lavaan R package (43).

## Results

### Self-Reported Adherence

At the 3 month follow-up, 61.4% (*n* = 27) reported having actively engaged in exposure maintenance; congruently, Poisson regression models revealed that these participants reported more (raw) in-between exposure occasions, both planned (B = 1.71, SE = 0.17, *p* < 0.001) and to a lesser extent involuntary (B = 0.79, SE = 0.25, *p* = 0.0018). Compliance groups did however not differ on post-treatment BAT (95% CI of mean difference: −0.45−2.49) or FSQ scores (95% CI of mean difference: −23.84−3.18). Somewhat fewer participants (43.2%, *n* = 19) reported having created a formal transition/maintenance plan, which in separate Poisson models was significantly associated with planned (B = 0.72, SE = 0.11, *p* < 0.001) but not involuntary exposure occasions (B = 0.31, SE = 0.22, *p* = 0.156). Among participants who created such a plan, average compliance was rated to M = 5.64 (SD = 1.95, IQR = 3). All but two participants (of 44) reported receiving a good rationale for exposure transition/maintenance, and 72.8% (*n* = 32) reported receiving sufficient instructions. Paired *t*-tests revealed that participants reported similar number of planned exposure occasions at the 3 and 12 month follow-ups (95% CI of mean difference: −7.43−0.73) yet more involuntary exposure occasions at the latter (t_40_ = −3.28, *p* = 0.0022), although it should be noted that covered periods are not equal in duration (3 vs. 9 months).

### *In-vivo* Exposures as Time-Varying Predictor

See Table 1 for full results (not including intercept and time predictor for each model). In no model and with neither outcome were involuntary exposure occasions associated with improvement. In all but one model (binarized exposure occasions predictor and BAT outcome) were planned exposure occasions independently associated with improvement.

**Table 1:** Independent impact of exposure frequencies in longitudinal models.

	**BAT**			**FSQ**		
**Predictor (frequency)**	**B**	**LB**	**UB**	**B**	**LB**	**UB**
Involuntary: raw	−0.03	−0.12	0.07	0.53	−0.6	1.95
Involuntary: binarized	−0.08	−1.74	1.03	4.18	−8.21	17.34
Involuntary: omitted	−0.03	−0.15	0.11	0.68	−0.96	2.28
Involuntary: truncated	−0.03	−0.13	0.09	0.66	−0.75	2.08
Planned: raw	0.03	0.00	0.07	−0.43	−0.83	−0.08
Planned: binarized	1.00	−0.24	2.23	−12.26	−22.68	−1.65
Planned: omitted	0.13	0.04	0.22	−1.66	−2.52	−0.78
Planned: truncated	0.09	0.02	0.17	−1.34	−2	−0.6

*Separate models that also included intercept and numeric time variable (not presented above)*.

### Sequential SEM

**Figure 2:**
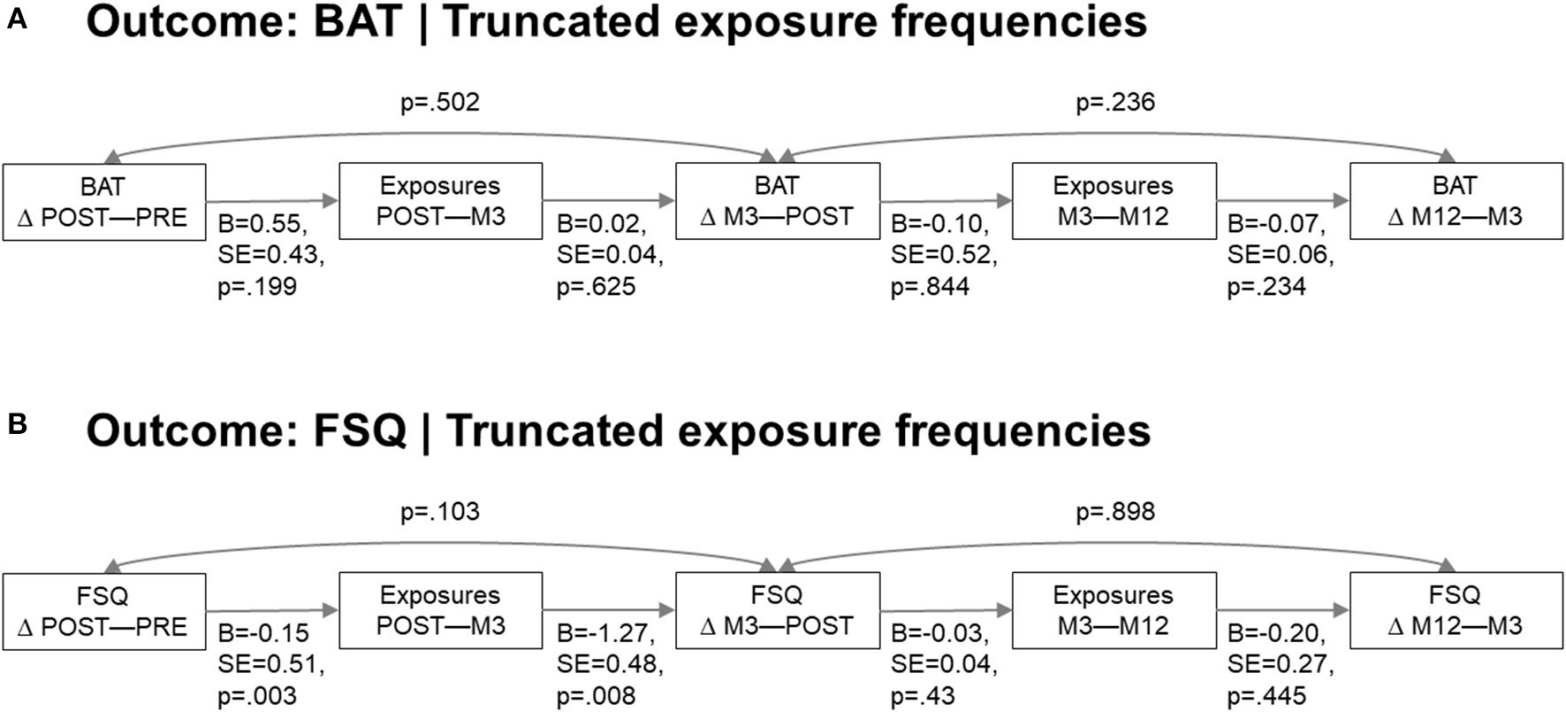
Sequential SEM. **(A)** Sequential model with BAT outcome. **(B)** Sequential model with FSQ outcome.

Since the results above showed that only planned exposures were associated with continue improvement, only this type of exposure was examined in sequential SEMs. There were no associations in any direction between temporally adjacent BAT score differences and planned exposures. However, congruent with the lowered threshold hypothesis, significant associations in the expected direction were found on FSQ scores in the first 3 months: a greater post-pre FSQ score difference (note negative value in case of improvement) was associated with more reported between-assessment exposure occasions at the 3 month follow-up, which in turn was associated with additional FSQ score decrease at this follow-up. However, sequential associations from here on were insignificant. See Figure 2 for full SEM results. A *post-hoc* analysis revealed that FSQ and BAT score differences did not correlate significantly (pre to post, *r* = 0.17, *p* = 0.246; post to 3 month, *r* = 0.145, *p* = 0.411), although a strong correlation remained between the two measures at each individual time point (all *p* < 0.002, *r* > −0.55), with negligible change in correlation strength over time (*r* = −0.58 pre-treatment, *r* = −0.55 at 12 month follow-up).

## Discussion

Meta-analytic research has shown that fear reduction following VR exposure therapy generalizes also to *in-vivo* equivalent stimuli; moreover, several studies report that individuals who have completed VR exposure therapy continue to improve in the year(s) that follow. The lowered threshold hypothesis proposes that this post-VR continued improvement is explained in part by individuals now being able to engage in *in-vivo* exposure in everyday life, breaking the vicious circle of avoidance that define phobias. The current study offers a first test of this hypothesis, the results of which are in partial support of the hypothesis: while reported planned (but not involuntary) *in-vivo* exposure occasions was independently associated with continued improvement over the course of 1 year (with one exception), a sequential model revealed the hypothesized bidirectional association between symptom decrease and extent of exposure applied only to self-reported phobia symptoms, not behavioral avoidance, and only during the first few months after treatment.

Finding no outcomes associations with number of involuntary exposures is expected given that this measure presumably captures life circumstances (e.g., type of residence and line of work) rather than treatment adherence, and was included to control for this possible source of bias. Associations with planned exposures were however largely robust across analyses and in the expected direction. While these findings need to be replicated in independent samples, our results are consistent with the observed difference in long-term outcomes across the two trials on this particular VR intervention (17, 18), and suggest that it is indeed beneficial to explicitly promote transition to *in-vivo* exposure after completing VR exposure, and that this can be accomplished with brief oral and written instructions. Even in the broader field of cognitive behavioral therapy (CBT), where numerous studies have examined predictors of relapse (44, 45), there has been very relatively little research on the associations between long-term symptom trajectories and post-treatment adherence to the therapeutic strategies taught and practiced during active treatment. This lack of comparable research also makes it difficult to interpret whether the self-rated compliance of 61% found in the current study is high or not. One rare previous study found that adherence to different CBT components for insomnia varied extensively between components, and that the most reportedly used component (74%) was in fact not associated with improvement, while behavioral strategies like stimulus control and sleep restriction (41% reported use) were (46). While such findings are obviously of great clinical interest, examining this particular research question entails facing several methodological barriers. First, in lieu of automatically collected measures using wearables (47), ecological momentary assessments (48), or even momentary reporting of involuntary spider exposures (49), measurements of adherence and/or exposure need likely be retrospective, at least to some temporal degree. Second, since adherence is typically not a parameter that participants can be randomly assigned to, special statistical techniques are required to estimate the causal effect of adherence in itself (50). Including a second-stage randomization after the VR intervention to either a transition component or not, with explicit adherence targets (frequency of exposures, habituation threshold etc.), would not only allow more advanced and appropriate statistical modeling, but would also resolve the need for outlier adjustment, as was the case in the current study.

Interestingly, binarized exposure counts were not significantly associated with BAT improvements and barely significantly associated with FSQ improvements. This suggests that one exposure occasion is not enough for continued improvement and that the numeric range (covered by the other variable version) is in itself clinically pertinent. A seemingly counter-intuitive finding was that immediate self-rated phobia symptom improvement, but not behavioral approach improvement, predicted number of subsequent *in-vivo* exposure exercises. This could be explained by individuals with great behavioral improvement seeing no need for further *in-vivo* exposure, and/or that improved performance for many came at the cost of high distress that discouraged further *in-vivo* exposure. The discrepancy between models, along with the corresponding weak correlation between improvement metrics (yet stable correlations at each time point) may also be due to a delay in improvement insight: a similar, reversed discrepancy—rapid behavioral improvement without simultaneous decrease in self-reported fear—has been observed in fear memory reconsolidation disruption experiments (51). Although we cannot rule out the influence of demand characteristics (52), the remarkable stability over time of the correlation between the objective and subjective outcome measures, suggests no or negligible effects only. It should also be noted that participants did not have access to prior FSQ nor were provided with any interpretation guidelines; further, since no further treatment was offered at follow-ups, participants had no incentive to exaggerate symptom ratings. Regardless of source of the diverging results, the findings of the current study shows the value of capturing both aspects of phobia presentation.

The sequential model revealed that planned exposure exercises did not influence continued symptom reduction (self-rated phobia symptoms specifically) beyond the 3 month follow-up, yet planned exposure exercises were associated with both types of symptom reduction in longitudinal models (with few exceptions) that included exposure exercises as a time-varying predictor, i.e., assuming equal effect of exposure exercises across time. While the former approach breaks down the presumed process in individual steps, the latter approach examines whether variance in observed outcomes that is not explained by time, is instead explained by frequency of exposure exercises. In addition, statistical power varies between approaches. Of interest, participants reported the same number of planned exposure exercises at the 3 and 12 month follow-ups, despite covering different durations, revealing that this became less common over time. However, over the same 12 months, treatment effects continued to grow (18), suggesting that other factors likely contribute to continued symptom improvement at a later stage. Examining a broader range of predictors of long-term outcomes in VR exposure therapy should be considered an important topic for future research.

### Strengths and Limitations

This first study on the lowered threshold hypothesis has several limitations that need to be acknowledged. First, the presence of outliers in the measure of post-VR exposures suggests some degree of measurement error. It should be noted that outlier here is used in the statistical sense, i.e., data points far off the observed distribution. All reported values were however in the plausible range (see Figure 1). Some measurement error with regards to what participants interpreted to constitute an exposure exercise is likely to have been present, and we cannot guarantee that some participants did not e.g., include exposure to television spiders. Future research would likely benefit from using more precise question phrasing. However, findings were largely consistent across outlier-adjustment methods, and many of the expected associations were found regardless, suggesting that these measures at least sufficiently captured what was intended. Second, exposures occasions were self-reported, retrospectively, and the temporal resolution was low. No auxiliary measures like experienced distress during and after each exposure task were collected either, nor was use of other therapeutic techniques (e.g., cognitive exercises) during the follow-up period measured. This should be considered an important goal of future research. Including continued VR exposure using applications specifically designed for at-home use (16, 20) would also allow collection of objective adherence data, yet would of course not cover the transition aspect. Using augmented reality technology to bridge VR and real-world exposure is a possible solution that remains to be explored. Third, the sample size and the lack of a comparison group randomized to not receive maintenance/transition promotion, precludes us from statistically estimating the causal effect of adherence to the transition program; this too should be addressed in future research. Of note, including also the *in-vivo* arm from the non-inferiority trial would not have address this issue and a comparison with other treatment modalities falls outside the scope of the current study, which focuses on the lowered threshold hypothesis for VR exposure therapy. Fourth, a consumer-targeted version of the same VR treatment was released in-between the 3 and 12 month follow-ups and the latter assessment did not include any question on use of this application. However, given the very limited consumer adoption of VR at this time, and the fact it was only available for one VR platform requiring a specific smartphone to run, the percentage of participants who had resumed VR exposure should be negligible if any.

Strengths of the study include the use of validated outcome measures, although as with any complex behavioral measure, we cannot rule out that equidistance in BAT scoring was suboptimal. Although results were not entirely consistent across outcome measures (see above for possible reasons), the use of two different statistical modeling techniques—with largely congruent findings—lends credibility. Other strengths include a low percentage of missing data at follow-ups, and that both the VR treatment and the subsequent promotion of *in-vivo* exposure were standardized.

## Conclusions

Our findings offer preliminary, partial support of the lowered threshold hypothesis of how Virtual Reality exposure therapy promotes continued symptom improvement. In longitudinal models with time-varying predictors, number of preceding *in-vivo* exposure occasions was associated with greater symptom decrease. In a sequential model, immediate post-treatment symptom reduction was associated with self-reported frequency of subsequent *in-vivo* exposures which in turn was associated with continued symptom reduction. However, this applied only to self-reported phobia symptoms, not behavioral avoidance, and was limited to the first 3 months of the follow-up. If these findings can be replicated in independent samples, it appears beneficial to actively promote *in-vivo* transition in order to maximize long-term effect of VR exposure therapy.

## Data Availability Statement

The raw data supporting the conclusions of this article will be made available by the authors, without undue reservation.

## Ethics Statement

The studies involving human participants were reviewed and approved by Stockholm Ethical Review Board. The patients/participants provided their written informed consent to participate in this study.

## Author Contributions

PL, PD, AM, GA, LR, WH, and PC designed the study. PL, PD, and WH collected the data. PL and PD analyzed the data. PL drafted the manuscript. PD, AM, GA, LR, WH, and PC revised the manuscript critically for important intellectual content. All authors contributed to the article and approved the submitted version.

## Conflict of Interest

WH is the founder and chief technology officer of Mimerse, a company specializing in developing VR interventions for mental health. PL has consulted for Mimerse but holds no financial stake in the company. The remaining authors declare that the research was conducted in the absence of any commercial or financial relationships that could be construed as a potential conflict of interest.
